# The effect of novel nitrogen-based chalcone analogs on colorectal cancer cells: Insight into the molecular pathways

**DOI:** 10.1016/j.heliyon.2024.e27002

**Published:** 2024-02-27

**Authors:** Arij Fouzat Hassan, Ola Hussein, Tara Al-Barazenji, Asma Allouch, Layla Kamareddine, Ahmed Malki, Ala‐Eddin Al Moustafa, Ashraf Khalil

**Affiliations:** a Department of Pharmaceutical Sciences, College of Pharmacy, QU Health, Qatar University, Doha, Qatar; b Department of Biomedical Science, College of Health Sciences, QU Health, Qatar University, Doha, Qatar; c Biomedical Research Centre, Qatar University, Doha, Qatar; d College of Medicine, QU Health, Qatar University, Doha, Qatar; e Oncology Department, McGill University, Montreal, QC, Canada

**Keywords:** Colorectal cancer (CRC), Chalcone, Nitrogen mustard, Methoxy, Analogs, Epithelial-mesenchymal transition (EMT)

## Abstract

In colorectal cancer (CRC), aberrations in *KRAS* are associated with aggressive tumorigenesis and an overall low survival rate because of chemoresistance and adverse effects. Ergo, complementary, and integrative medicines are being considered for CRC treatment. Among which is the use of natural chalcones that are known to exhibit anti-tumor activities in *KRAS* mutant CRC subtypes treatment regimens. Consequently, we examine the effect of two novel compounds (DK13 and DK14) having chalcones with nitrogen mustard moiety on CRC cell lines (HCT-116 and LoVo) with *KRAS* mutation. These compounds were synthesized in our lab and previously reported to exhibit potent activity against breast cancer cells. Our data revealed that DK13 and DK14 treatment suppress cell growth, disturb the progression of cell cycle, and trigger apoptosis in CRC cell lines. Besides, treatment with both compounds impedes cell invasion and colony formation in both cell lines as compared to 5-FU; this is accompanied by up and down regulations of E-cadherin and Vimentin, respectively. At the molecular level, both compounds deregulate the expression and phosphorylation of β-catenin, Akt and mTOR, which are the main likely molecular mechanisms underlying these biological occurrences. Our findings present DK13 and DK14 as novel chemotherapies against CRC, through β-catenin/Akt/mTOR signaling pathways.

## 1 Introduction

The incidence of colorectal cancer (CRC) has been rapidly rising across the globe [1]. In 2020, 0.94 million CRC-related fatalities occurred globally, accounting for 9.4% of all cancer-related deaths and 10% of the world's cancer incidence [2]. Being one of the most aggressive cancers, CRC ranks as the world's second most lethal form of cancer [3]. The onset of CRC is predominantly linked to environmental factors such as dietary habits, physical activity, smoking, and alcohol consumption [4].

At the molecular level, sporadic mutations account for 70% of CRC cases, with familial (25%) and inherited mutations (5–10%) accounting for the remaining 30% of cases [5].Various genetic and epigenetic alterations, particularly those that impact the expression of tumor suppressor genes (TSGs), such as adenomatous polyposis coli (APC), play vital roles in the progression towards malignancy, as it is manifested in 85% of CRC cases [6,7]. *KRAS* aberrations are associated with 30–60% of sporadic CRCs [8], and oncogenic *KRAS* is responsible for adenoma to carcinoma transition and subsequent activation of proto-oncogenes that regulate cellular proliferation and differentiation [9,10]. Prior studies have revealed a correlation between *KRAS* mutation and increased rates of metastasis, recurrence, and mortality among patients with CRC [[11], [12], [13]]. Under normal conditions of cellular differentiation and proliferation, the activation of the EGFR pathway initiates multiple downstream intracellular signaling pathways, including the RAS/RAF/MEK/ERK, PI3K/AKT, and JAK/STAT3 pathways [14,15]. EGFR alterations have been documented in diverse cancer types, including CRC [16]. Distorted EGFR signaling pathway contribute immensely to CRC initiation, progression, and dissemination via PI3K/AKT/mTOR pathway [17]. These are responsible for the upregulation of survival signals and escaping from apoptosis in CRC [18]. Further, it is well documented that there is an interaction between PI3K/AKT/mTOR and Wnt/β-catenin pathways that is critically involved in CRC progression and tumorigenesis. The relationship between both pathways makes them a unique target for therapeutic strategies to treat CRC [19,20]. Various studies have; however, reported that *RAS*mutant subtypes are resistant to anti-EGFR treatments beside the absence of Wnt inhibitor in clinical practice [[21], [22], [23], [24]], necessitating the development of novel and effective therapeutic compounds against *KRAS* mutant CRCs as an alternative.

One of the highly attractive scaffolds that are currently being investigated as potential multitargeted anticancer agents is chalcone. Due to their fascinating biological properties, chalcones-rich plants were historically used in traditional medicine. Chalcones, also known as benzylideneacetophenone, are open-chain flavonoids that are widely distributed in various plant species [25]. Chalcones demonstrate a broad spectrum of biological activities, including antidiabetic [26], anti-inflammatory [27], antimicrobial [28], antioxidant [29], antihypertensive [30] and anticancer [31]. For instance, Metochalcone, a natural chalcone isolated from the heartwood of *Pterocarpus marsupium*, was approved for use as a choleretic and diuretic agent [32]. Similarly, sofalcone, a natural chalcone found in *Sophora tonkinensis*, was marketed as an anti-ulcer agent [33,34]. The variety in biological activity stems from the unique features of the chalcone skeleton that allow a large number of derivatives to be generated that differ in their specificity and reactivity with biological targets [35]. Besides, chalcones have long fascinated medicinal chemists due to their ease of synthesis, poor interaction with DNA and low risk of mutagenicity [36,37].

Various natural and synthetic-derived chalcones have shown promising anticancer activities, attributed to their capacity to inhibit various CRC targets such as the AKT/mTOR and Wnt/β-catenin pathways [[38], [39], [40]]. Some chalcone-based compounds have been shown to also exhibit proapoptotic and cytotoxic effects and modulatory functions, presenting chalcone compounds as potential therapeutic agents against cancers, including CRC [41]. Indeed, various studies have reported an anti-cancer activity of chalcones and their derivatives [31,42,43]. However, no chalcone had been moved into subsequent developmental steps for the treatment of CRC. This could be partially attributed to their modest potency or lack of *in vivo* activity.

Recently, we developed two novel chalcone-based compounds bearing a nitrogen mustard moiety (DK13 and DK14) that showed promising anticancer activity against HER-2 positive and triple-negative breast cancer (TNBC) cell lines [31,42]. Interestingly, DK14 demonstrated a potent *in vivo* activity in a mice xenograft model, which presents these compounds as a potential alternative therapy to the most aggressive breast cancer subtypes. As such, we aim in this study to assess the effectiveness of DK13 and DK14 on *KRAS* mutant CRC *in vitro* using HCT116 and LoVo cell lines and to identify their molecular targets.

## 2 Material and methods

### 2.1 Cell culture

HCT-116 and LoVo cell lines were purchased commercially from the American Type Tissue Culture (ATCC) (USA). The cells were cultured in complete cell culture media, Gibco® Dulbecco's modified Eagle medium (DMEM) (Gibco, Life Technologies, Massachusetts, MA, USA), supplemented with 10% Fatal Bovine Serum (FBS) (PAN-Biotech, Aidenbach, Bavaria, Germany), and 1% penicillin/streptomycin (1X) antibiotics (Thermo Fisher Scientific, USA). The cells were incubated at 37 °C in a 5% CO_2_ environment.

### 2.2 Cell viability assay

CRC cell lines (HCT-116 and LoVo) were seeded in 96-well plates at a density of 5000 cells per well and incubated for 24 h. Following this, the old media was replaced with fresh media. The CRC cell lines were then subjected to various concentrations (2.5 μM, 5 μM, 10 μM, 20 μM, 40 μM, and 60 μM) of DK13 and DK14 compounds for a duration of 48 h. For comparison, untreated cells were cultured in 100 μl of media, and cells treated with DMSO only served as controls. After 48 h, AlamarBlue™ cell viability reagent (Invitrogen, Thermo Fisher Scientific, Waltham, MA, USA) diluted in media (1:10) was added to each well and incubated for 3 h. Fluorescence measurements were recorded using a Tecan fluorescent plate reader (Infinite M200, Tecan, Grödig, Austria) with an excitation wavelength of 560 nm and an emission wavelength of 590 nm. Cell viability was determined by calculating the fluorescence intensity of DK13 and DK14-treated cells relative to the control cells.

### 2.3 Morphological examination

CRC cells were seeded in 6-well plates at a density of 2 x 10^5^ cells per well and allowed to incubate for 24 h. The following day, the old media was discarded, and the cells were treated with 10 μM of DK13, DK14, and 5-Fluorouracil (5-FU) (used as a positive control) for a duration of 48 h. As for the control groups, fresh media was added and treated with DMSO. The cell morphology was assessed using inverted phase-contrast microscopy with a 10× objective lens and captured using the LEICA MC170 HD camera from Leica Microsystems in Wetzlar, Germany.

### 2.4 Colony formation

To assess the cells' capacity to form colonies *in vitro*, a soft agar assay was conducted following established protocols [44]. In brief, HCT-116 and LoVo cells (1 x 10^4^ cells/well) were suspended in DMEM medium containing 0.3% agar. For treated cells, DK13 (10 μM) or DK14 (10 μM) was included, while control cells received no treatment. The cell suspension was then plated over a layer of DMEM medium containing 10% FBS and 0.4% agar. The culture was monitored every 2–3 days for a duration of 4 weeks to observe colony formation. Multiple locations within each well were photographed using an inverted light microscope from Leica in Wetzlar, Germany to capture images of the colonies.

### 2.5 Flow cytometric analysis of cell cycle

For the experiment, CRC cells were seeded in 100 mm Petri dishes at a density of 8 x 10^5^ cells per dish and incubated overnight. To synchronize the cells in the G0 phase of the cell cycle, they were starved with serum-free DMEM medium for 24 h.

Following synchronization, the cells were treated with 10 μM of DK13, DK14, and 5-FU for a duration of 48 h. Subsequently, the cells were harvested, washed twice with Phosphate Buffer Saline (PBS), and fixed overnight in 70% ice-cold ethanol. To stain the DNA, the cells were treated with FXCycle PI/RNase staining solution (Invitrogen, Thermo Fisher Scientific) after undergoing RNase A treatment at 37 °C for 30 min. Cell-cycle analysis was performed using flow cytometry (BD Accuri C6, BD Biosciences, USA), and the FlowJo software was used to quantify the cells in the G0/G1, S, and G2/M phases.

### 2.6 Invasion assay

Corning™ BioCoat™ Matrigel™ Invasion Chamber (8 μm; Fisher Scientific) was utilized following the manufacturer's instructions to perform the invasion assay. In brief, the bottom chamber of the invasion chamber was filled with DMEM medium supplemented with 10% FBS. The upper chamber was seeded with treated and untreated cells (5 x 10^4^ cells) in serum-free DMEM media. The chamber was then placed in a CO_2_ incubator at 37 °C. After 48 h of incubation, non-invasive cells on the upper surface of the chamber were removed by gently scraping with a cotton swab. The cells that successfully invaded the lower chamber were fixed using methanol and stained with 0.4% crystal violet followed by DAPI staining. For quantification, the invaded cells were counted in five pre-determined fields under a fluorescence microscope (Leica DMi1, Leica Microsystems, Wetzlar, Germany). The percentage of inhibition of invaded cells was calculated by comparing it to the number of invaded cells in the untreated (control) group.

### 2.7 Western blot analysis

To evaluate the expression levels of target genes, Western blot analysis was conducted. Initially, CRC cells were seeded in 100 mm dishes at a seeding density of 1.5 x 10^6^ cells per well and incubated for 24 h. On the following day, the old media was replaced with fresh media containing 10 μM of DK13, DK14, 5-FU, or DMSO as a control, and the cells were further incubated for 48 h. Protein lysates were extracted from the treated and control cells using a 10% SDS lysis buffer, and the protein concentration was determined using the Pierce BCA protein assay kit (Pierce Biotechnology, Rockford, IL, USA). Thirty micrograms of protein from each treatment group were separated on gradient polyacrylamide gels (PAGE) and transferred onto PVDF membranes. The membranes were then probed with specific primary antibodies, including anti-rabbit E-Cadherin (Cell Signaling Technology), anti-mouse Vimentin (Thermo Fisher Scientific), anti-rabbit β-Catenin (Cell Signaling Technology), anti-rabbit Phospho-β-Catenin (Cell Signaling Technology), anti-rabbit Akt, anti-rabbit Phospho-Akt (Cell Signaling Technology), anti-rabbit mTOR (Abcam, Cambridge, MA, USA), and anti-rabbit Phospho-mTOR (Cell Signaling Technology). For quantification of protein expression, the band intensities were normalized by re-probing the blots with anti-rabbit GAPDH (Abcam: abID# ab9485). Chemiluminescence ECL-Western blotting (Pierce Biotechnology, Waltham, MA, USA) was employed to detect the protein bands according to the manufacturer's instructions. The protein bands were then quantified using ImageJ software, and their intensities were normalized to that of GAPDH.

### 2.8 Statistical analysis

Statistical analysis and graphical representation of the results were performed using the Graph Pad Prism9 software. The IC_50_ values were determined by employing a non-linear regression equation generated in Microsoft Excel. To assess the statistical significance between the treated and control groups, a one-way ANOVA followed by Tukey's post-hoc test was conducted. The results were expressed as mean ± standard error of the mean (SEM) with a sample size of 3 for all experiments.

## 3 Results

**Fig. 1:**
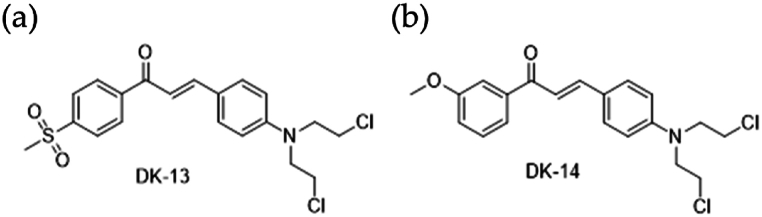
Chemical structure of compounds (a) DK13 and (b) DK14.

**Fig. 2:**
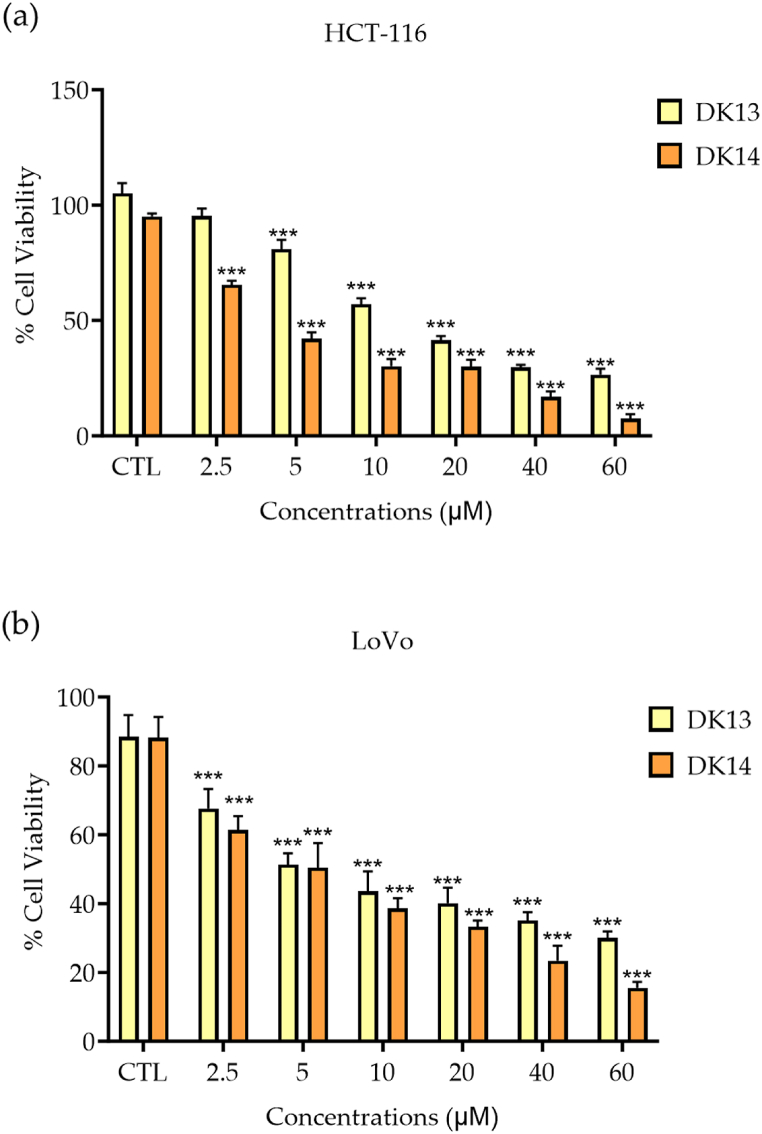
Effect of DK13 and DK14 chalcone compounds treatment on the viability of (a) HCT-116 and (b) LoVo cell lines. Cell viability relative to control (DMSO) was measured by AlamarBlue after 48 h of treatment. Data are presented as Mean ± SEM of three independent experiments. One-way ANOVA followed by Tukey's post-hoc test was used to compare the treatment groups. Results were considered statistically significant when *p<0.05*. ***p < 0.001.

To evaluate the effect of DK13 and DK14 chalcone compounds (Fig. 1 a &b) on *K-RAS* mutant CRC, two CRC cell lines with *K-RAS* mutations, HCT-116 and LoVo, were treated with increasing concentration (2.5 μM, 5 μM, 10 μM, 20 μM, 40 μM and 60 μM) of DK13 and DK14 compounds for 48 h. As anticipated, treatment with both DK13 and DK14 compounds significantly reduced the viability of HCT-116 (Fig. 2a) and LoVo (Fig. 2b) CRC cell lines in a dose-dependent manner. Based on our findings herein and on those recently reported in our published work on the effect of DK13 and DK14 on TNBC and HER2-positive breast cancer, the IC_50_ for both cell lines, which is close to 10 μM was selected to conduct all further investigation on both CRC cell lines [31,42]. Further, it was reported by Wang et al. (2019), the IC_50_ values of 5-FU are 11.23 μM and 12.28 μM for HCT-116 and LoVo cell lines, respectively [45]. These values are close to the IC_50_ of DK13 and DK14, therefore 10 μM of 5-FU was used in all investigations as a positive control.

**Fig. 3:**
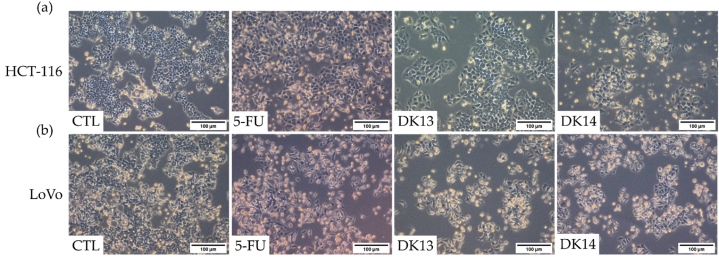
Effects of DK13 and DK14 chalcone compounds treatment on (a) HCT-116 and (b) LoVo cell lines morphological features. Cells were treated with 10 μM of 5-FU, DK3 and DK14. Treatment with 10 μM DK13 and DK14 for 48 h induce the formation of a monolayer of cells and increase cell-cell adhesion tendency as compared to that detected in untreated and 5-FU-treated cells, which display a round phenotype and form multilayers. Images were taken using a 10× objective (N = 3). The scale bar represents 100 μm.

Next, the effect of 10 μM DK13 and DK14 treatment for 48 h on HCT-116 and LoVo cells morphological features in comparison to the effect of DMSO and 5-FU (positive control) on treated cells was determined. As shown in Fig. 2, in the absence of DK13 and DK14 treatment, HCT-116 and LoVo cells exhibit a round morphology and disorganized multilayered cells. Upon DK13 and DK14 treatment, however, the morphology of both cell lines changed from a mesenchymal phenotype to an epithelial-like phenotype with an increase in their cell-cell adhesion, where cells became relatively flattened in shape. While some treated cells displayed loss of membrane integrity, cell shrinkage, indicating cell death. The suggested induction of round to epithelial-like transition (RELT) pattern upon DK13 and Dk14 treatment was not observed in cells treated with 5-FU (Fig. 3).

**Fig. 4:**
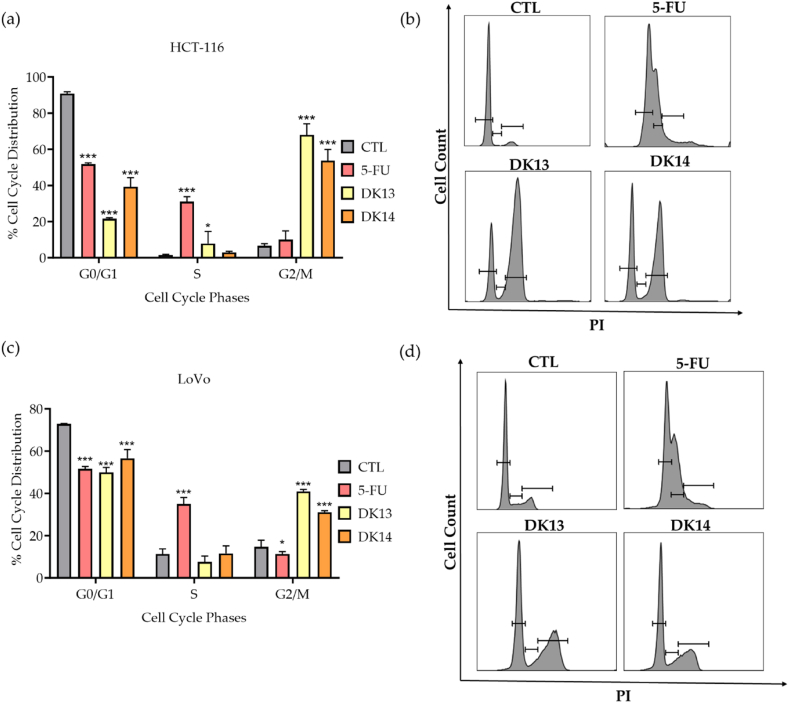
Effect of DK13 and DK14 chalcone compounds treatment on cell-cycle phase distribution in (a,b) HCT-116 and (c,d) LoVo cell lines. Flow cytometry analysis and histograms presenting the G0/G1, S-phase, and G2/M cell cycle phases in cells treated with DK13 and DK14, then stained with propidium iodide as compared to those treated with DMSO and 5-FU. Data are presented as Mean ± SEM of three independent experiments. One-way ANOVA followed by Tukey's post-hoc test was used to compare the treatment groups. Results were considered statistically significant when *p<0.05*. *p<0.05 and ***p < 0.001.

To further validate the effect of DK13 and DK14 treatment on CRC cell lines proliferation, the cell-cycle phase distribution of cells treated with each chalcone compound was analyzed by flow cytometry. As shown in Fig. 4, DMSO-treated cells displayed a typical cell cycle pattern in all phases. In contrast, treatment of both HCT-116 and LoVo with 10 μM DK13 and DK14 induced a significant cell cycle arrest at the G2/M phase. On the other hand, 5-FU treatment arrested both cell lines at the S-phase.

**Fig. 5:**
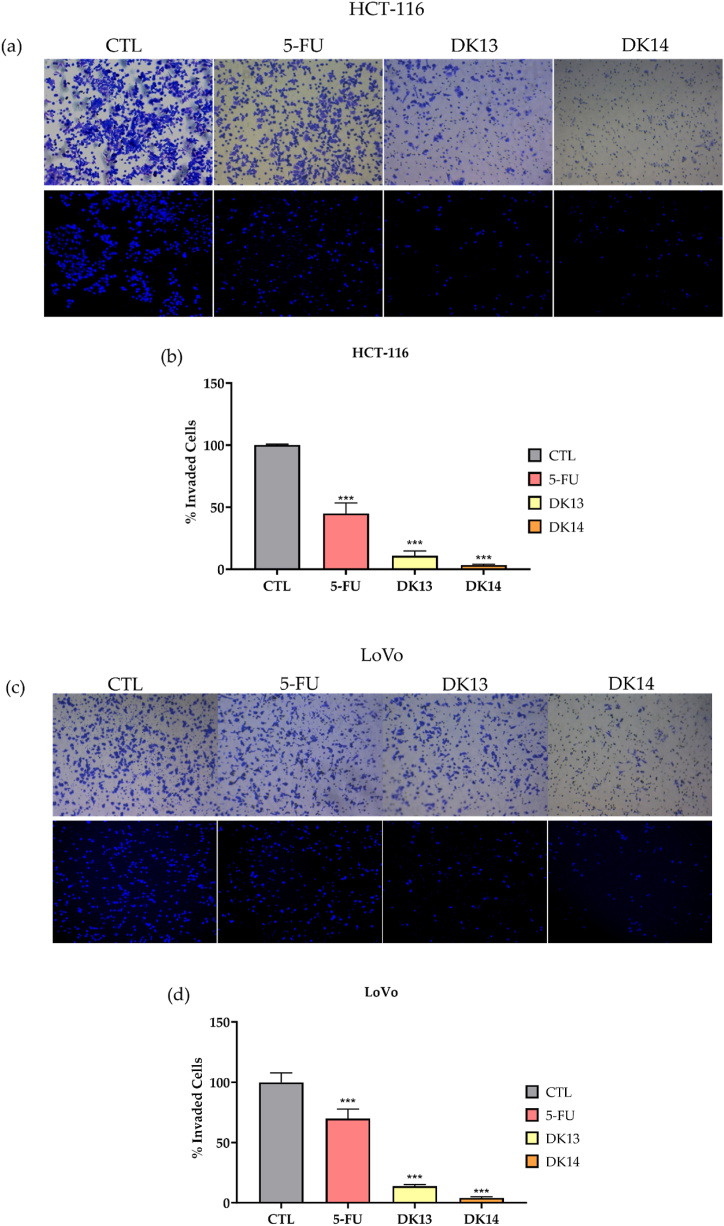
Effect of DK13 and DK14 chalcone compounds on cell invasion ability of (a,b) HCT-116 and (c,d) LoVo cell lines. Treatment with 10 μM DK13 and DK14 for 48 h, significantly inhibited the cell invasion ability of HCT-116 and LoVo cells as compared to DMSO, and 5-FU treated cells. Data are presented as Mean ± SEM of three independent experiments. One-way ANOVA followed by Tukey's post-hoc test was used to compare the treatment groups. Results were considered statistically significant when *p<0.05*. ***p < 0.001. Images were taken using a 10× objective (N = 3). The scale bar represents 100 μm.

Subsequently, and to examine the effect of DK13 and DK14 on inhibiting CRC cells invasion ability, a Matrigel invasion assay was carried out. Treatment with 10 μM DK13 and DK14 significantly inhibited the invasion ability of HCT-116 cells by 89.2 % and 96.7 %, respectively and of LoVo cells by 86% and 95.8%, respectively as shown in Fig. 5. On the other hand, treatment with 5-FU inhibited cell invasion by 55% and 30% in HCT-116 and LoVo cells, respectively (Fig. 5).

**Fig. 6:**
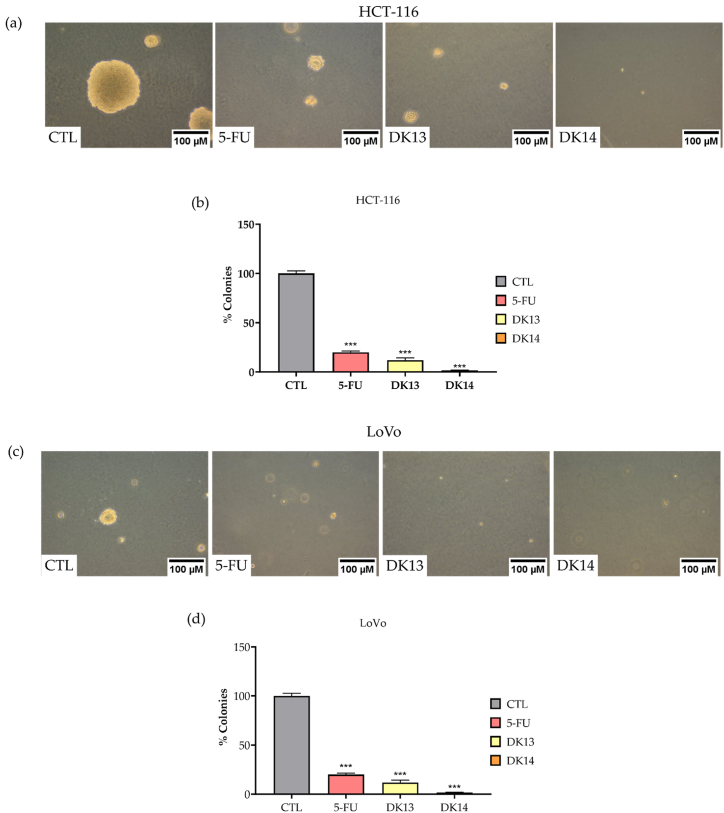
Effect of DK13 and DK14 chalcone compounds on colony formation ability of (a,b) HCT-116 and (c,d) LoVo cells. Treatment with10μM DK13 and DK14 for 48 h significantly inhibits the colony formation ability of both HCT-116 and LoVo cells on soft agar as compared to DMSO and 5-FU treated cells. Data are presented as Mean ± SEM of three independent experiments. One-way ANOVA followed by Tukey's post-hoc test was used to compare the treatment groups. Results were considered statistically significant when *p<0.05*. ***p < 0.001. Images were taken using a 10× objective (N = 3). The scale bar represents 100 μm.

We further examined the effect of DK13 and DK14 treatment on the ability of HCT-116 and LoVo cells to form colonies in soft agar for a period of two weeks. As compared with their matched controls, our data revealed a significant reduction in the number of HCT-116 and LoVo cells colonies when treated with each chalcone compound (Fig. 6). For HCT-116 cells, the number of colonies was significantly reduced by 88% and 98%, upon DK13 and DK14 treatment, respectively (Fig. 6 a,b). Similarly, for LoVo cells, the number of colonies was significantly reduced by 78.5% and 98%, respectively, as compared to untreated cells (Fig. 6 c,d). Interestingly, these findings advocate the role of chalcone compounds in inhibiting colony formation, a hallmark of tumor growth *in vivo*.

**Fig. 7:**
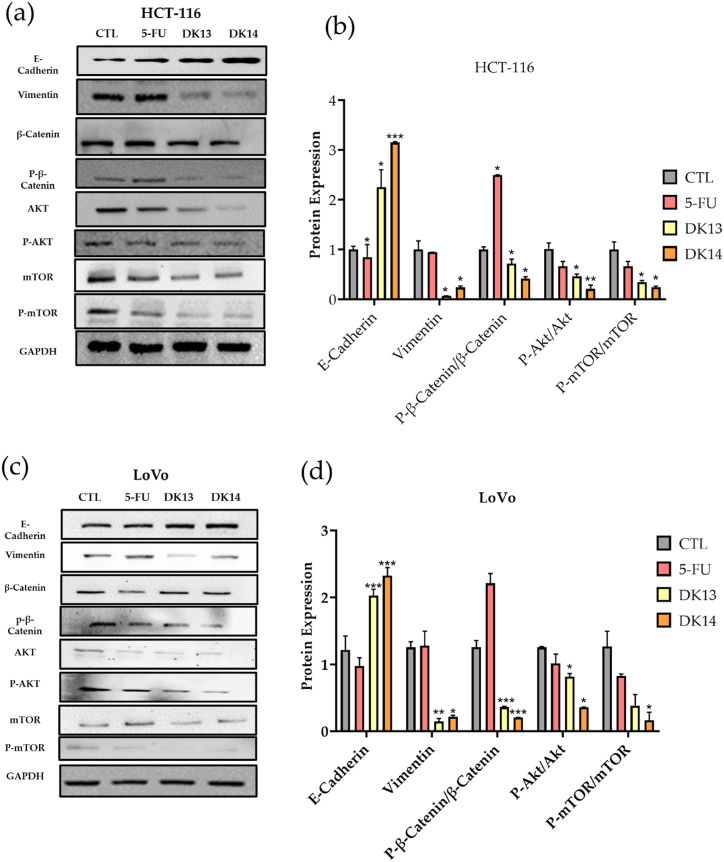
Effect of DK13 and DK14 chalcone compounds on the expression patterns of EMT biomarkers and Akt/mTOR in (a,b) HCT-116 and (c,d) LoVo cell lines. After 48 h of 10 μM DK13 and DK14 treatment, an overexpression of E-cadherin, and a downregulation of vimentin, p-β-catenin, Akt, *p*-Akt, mTOR, *p*-mTOR as compared to DMSO-treated and 5-FU treated cells was detected. GAPDH serves as loading control. Data are presented as Mean ± SEM of three independent experiments. One-way ANOVA followed by Tukey's post-hoc test was used to compare the treatment groups. Complete, unadjusted images are included in the supplemental material. Results were considered statistically significant when *p<0.05*. *p<0.05, **p<0.01 and ***p < 0.001.

Collectively, our obtained data suggest an effect of DK13 and DK14 on the deregulation of cell morphology, colony formation and inhibiting cell invasion. As such, we examined the impact of DK13 and DK14 treatment on the expression of key players involved in the epithelial-mesenchymal transition (EMT) pathway, which is a major hallmark of cell invasion and metastasis, in HCT-116 and LoVo cell lines using Western blot analysis. As expected, our findings revealed that treatment with 10 μM DK13 and DK14 compounds causes an increase in the expression patterns of E-cadherin, and a decrease in that of vimentin and p-β-catenin in both CRC cell lines, which are major biomarkers of EMT, as shown in Fig. 7.

Additionally, since *KRAS* mutation stimulates the activation of the Akt/mTOR pathway leading to unregulated cell proliferation and survival [46], and given the previously reported role of the Akt/mTOR pathway in regulating cell proliferation, EMT progression, cell invasion, and colony formation in *KRAS* mutant CRC cells [47,48], we anticipated an Akt/mTOR pathway-dependent anti-cancer effect of DK13 and DK14. As such, we explored the effect of 10 μM DK13 and DK14 on the expression patterns of Akt and mTOR proteins. As hypothesized, DK13 and DK14 treatment inhibited the phosphorylation of Akt and mTOR in *KRAS* mutant CRC cells (Fig. 7). Taken together, our data suggest that both DK13 and DK14 chalcone compounds deregulate EMT biomarkers by acting on β-catenin and Akt/mTOR pathways in both CRC cell lines as evident by the significant deregulation of the phosphorylated forms of β-Catenin, Akt and mTOR proteins in DK13 and DK14 treated cells as compared to their matched controls.

## 4 Discussion

In this study, we investigated the effect of two nitrogen-based chalcone analogs, DK13 and DK14 on cell proliferation, cell cycle progression, cell invasion, and colony formation ability in HCT-116 and LoVo *KRAS* CRC cell lines. The two nitrogen-based chalcone analogs used in this study were synthesized through Claisen-Schmidt condensation reaction and used at a 10 μM concentration in a previous study conducted by our team on TNBC and HER2-positive breast cancer cell lines. Our previous findings in breast cancer cells revealed a potent ability of DK13 and DK14 treatment to significantly suppress cell proliferation and division ability [31,42]. These results support our findings in this study on CRC cell lines; yet with a more pronounced effect of DK14 as compared to that of DK13 on both cell lines. This can be due to the methoxy substitution present in DK14 as compared to DK13 that carries a 4-methylsulfonyl substitution [31]. The localization of the methoxy group in the meta position of the ring seems critical for the anti-cancer activity of DK14. Various studies have reported the role of the methoxy group in augmenting the anticancer activity of chalcone compounds [[49], [50], [51]]. A previous study conducted on Q705, a benzylidene tetralones cyclic chalcone analogue, had also revealed a similar effect on HCT-116 cell proliferation as the one detected in this study [52]. Likewise, and in concordance with our findings, novel poly-methoxylated chalcones (compounds 3 and 14) were also shown to inhibit HCT-116 and LoVo cell lines proliferation [22].

Along this, the effect of DK13 and DK14 on inducing HCT-116 and LoVo cells morphological changes by mesenchymal to epitheliallike transition (MET) aligns with our previous work on DK13 and DK14 using different types of cancer cells [31,42]. In addition, growth inhibition of both cell lines was clearly observed upon treatment with DK13 and DK14 compared to the control. These observed morphological changes were also accompanied by an increase in the expression of E-cadherin and a decrease in the expression of vimentin and p-β-catenin. E-cadherin is known to suppress the progression and invasion of CRC [53], while loss of E-cadherin is highly associated with CRC metastasis [54]. On the other hand, vimentin and p-β-catenin are highly expressed in invasive tumors including CRC cells and are negatively associated with CRC patients’ survival [[55], [56], [57], [58]]. High expression of vimentin in CRC influences metastasis and promotes resistance to current anti-cancer therapies [59]. similarly, activation of β-catenin is well known to play a critical role in facilitating tumor chemoresistance [60,61]. Although 5-FU serves as the first-line therapy for CRC, previous studies have shown no significant effect of 5-FU on the expression of vimentin [62]. Further, it was reported that 5-FU induces CRC stemness through β-Catenin activation [63]. Based on our findings, 5-FU had no effect on the expression of vimentin, while it significantly upregulated the expression patterns of p-β-catenin. Interestingly, treatment with our novel chalcone compounds decreased vimentin and β-catenin expression patterns, presenting DK13 and DK14 as more potent therapeutic agents as compared to 5-FU for CRC. Additionally, the E-cadherin/β-catenin adhesion complex dissociation is essential for EMT in leading to CRC invasion and metastasis [64].

Moreover, the detected growth inhibition of HCT-116 and LoVo cells proliferation can be explained more by the G2/M accumulation detected in DK13 and DK14 treated cells. Similar to our findings, a previous study reported that chalcones suppress cyclin D1 and induce p53 expression leading to G2/M phase arrest [65]. Likewise, a similar effect on cell cycle distribution was also observed in HCT-116 cells treated with Q705 [52]. Furthermore, C1 and C2, 2′-hydroxy chalcone derivatives, were shown to affect HCT-116 cell cycle distribution by increasing the percentage of CRC cells count in G2/M phase as compared to normal control [66]. It has been also reported that HMNC-74 treatment triggers colon cancer cell line, SW620, cycle arrest at G2/M phase [67]. Moreover, several studies reported the potentiality of methoxy-chalcone derivatives in damaging the DNA through cell cycle disturbance in different cancer types including melanoma, Leukemia, non-small cell lung, breast, cervical and gastric cancers [[68], [69], [70], [71]]. However, the effect of DK13 and DK14 on cell cycle distribution in TNBC and HER-2 positive cancer cells was different, as both compounds caused S phase blockade in both cancer types [31,42]. The effect of DK13 and DK14 on the cell cycle could be potentially related to the chemical structure of these compounds as the nitrogen mustard group present in both compounds can act as an alkylating agent that binds to the DNA and prevents replication of the genetic material [72]. Along this, our findings also revealed that DK13 and DK14 treatment inhibits HCT-116 and LoVo cells division and invasion abilities, a finding congruent to that reported in our recently published work on the effect of DK13 and DK14 treatment on TNBC and HER2-positive breast cancer cells [31,42]. Previous findings have also reported that the KB-34 Chalcone (1,3-diphenyl-2-propen-1-one) derivative inhibits HT-29 and SW620 CRC cell lines invasion ability [73]. Also, the L2H17 chalcone analog was reported to suppress invasion and migration of mouse CT26.WT colon cancer cells [74].

Furthermore, we analyzed the effect of DK13 and DK14 treatment on the expression of Akt/mTOR. As expected, our data show that DK13 and DK14 treatment significantly down-regulates the expression of Akt and *p*-Akt as well as mTOR and *p*-mTOR in HCT-116 and LoVo cells. The Akt/mTOR pathway plays a critical role in colorectal carcinogenesis by initiating metastasis and tumor progression [[75], [76], [77]]. Additionally, they are considered a key component in regulating the EMT pathway by downregulating E-cadherin, RhoA and Rac1 signaling [48,78]. This is supported by our findings that revealed the effect of DK-chalcone compounds on inhibiting cell invasion and blocking colony formation of both CRC cell lines. Likewise in breast cancer and esophageal cancer cells, chalcones inhibit cell growth, invasion, and migration by reinstating the E-cadherin/catenin complex and impeding Wnt/β-catenin signaling [79,80]. In the present study, as mentioned above, we report that the inhibition of β -catenin activation plays an important role in decreasing the cell invasion ability of CRC cells, which is in concordance with these studies.

Taken together, our findings present DK chalcone-based compounds as potential therapeutic agents for CRC and opens the door for future *in vivo* studies directed towards determining the impact of DK13 and DK14 on tumor growth using orthotopic xenograft and/or transgenic mouse models and for explicit mechanistic studies to conclude DK13 and DK14 targeted signaling pathway.

## 5 Conclusions

In conclusion, we demonstrate herein the anti-tumour effect of two chalcone-based compounds, DK13 and DK14, on CRC *KRAS* mutant cell lines, HCT-116 and LoVo, and provide some insight on the plausible signalling pathway targeted by these two compounds. In an effort to develop novel targeted therapies for CRC, and building upon our prior work on chalcone-based nitrogen compounds targeting breast cancer, we highlight the potential of compounds DK13 and DK14 as promising therapeutic candidates for CRC, since they target β-catenin, Akt and mTOR signalling pathways, which play crucial roles in colorectal carcinogenesis. Besides, our study revealed that these compounds, suppress the proliferation, arrest the cell cycle at the S phase and trigger apoptosis of CRC cells. Moreover, both compounds had remarkable inhibition in colony formation and cell invasion, which was superior to 5-FU, a first-line chemotherapy. In concordance with the effect previously found in TNBC cells, compound DK14 was recognized as the most promising candidate for further investigation against CRC as compared to results found in HER2-positive breast cancer cells, which identified DK13 as the most promising compound. Based on our findings, further investigations to unravel the detailed mechanism of DK13 and DK14 action in CRC are warranted. More importantly, given their versatility against highly aggressive cancer types, the effect of these compounds on other types of human carcinomas can be the focus of future studies. Thus, these novel chalcone compounds may pave the way for advanced therapeutic approaches in human CRC management, especially in *KRAS* mutant CRC, as well as other cancer types.

## Funding

This research was funded by 10.13039/501100004252Qatar University internal grants and QNRF: QUCP-CMED-22/23–529 and QUCG-CMED-20/21-2, QUCG-CPH- 22/23–510 and UREP28-022-3-005.

## Data availability statement

The original contributions presented in the study are included in the article, further inquiries can be directed to the corresponding author/s. Data will be made available on request.

## CRediT authorship contribution statement

**Arij Fouzat Hassan:** Writing – original draft, Validation, Methodology, Formal analysis, Data curation. **Ola Hussein:** Writing – original draft, Methodology, Formal analysis, Data curation. **Tara Al-Barazenji:** Methodology. **Asma Allouch:** Methodology. **Layla Kamareddine:** Writing – review & editing, Supervision. **Ahmed Malki:** Writing – review & editing, Supervision. **Ala‐Eddin Al Moustafa:** Writing – review & editing, Writing – original draft, Validation, Supervision, Resources, Funding acquisition, Data curation, Conceptualization. **Ashraf Khalil:** Writing – review & editing, Writing – original draft, Validation, Supervision, Resources, Funding acquisition, Data curation, Conceptualization.

## Declaration of competing interest

The authors declare that they have no known competing financial interests or personal relationships that could have appeared to influence the work reported in this paper.
